# General or Vocational Education? The Role of Vocational Interests in Educational Decisions at the End of Compulsory School in Switzerland

**DOI:** 10.1007/s12186-020-09256-y

**Published:** 2020-08-15

**Authors:** Andreas Jüttler, Stephan Schumann, Markus P. Neuenschwander, Jan Hofmann

**Affiliations:** 1 Department of Economics, University of Konstanz, Universitätsstraße 10, 78457 Konstanz, Germany; 2 Center for Learning and Socialization, University of Applied Sciences and Arts Northwestern Switzerland, Obere Sternengasse 7, CH-4502 Solothurn, Switzerland

**Keywords:** Vocational interests, RIASEC, Career decisions, Vocational training, Upper secondary education, General education

## Abstract

Many educational systems are characterized by segregation between a general and vocational educational track. When adolescents must decide on their postcompulsory education at the end of lower secondary school, the different programs are typically embedded in one of these two main tracks. Prior career choice theories postulate that vocational interests, as structured by the six-dimensional RIASEC model of Holland (1997), play a crucial role in educational and vocational transition processes. However, regarding the question of general versus vocational education, previous studies have mainly focused on the effects of social background. Therefore, this paper examines the impact of vocational interests on the choice of Baccalaureate School (BAC, general track), Vocational Education and Training (VET, vocational track) or the Federal Vocational Baccalaureate (FVB), a hybrid qualification that links elements of both tracks. The sample consists of *N* = 609 students at the end of lower secondary school in Switzerland. The results of multinomial logistic regression analyses show that all six dimensions of Holland’s interest model are significant predictors for the three postcompulsory tracks, even when controlling for school variables (e.g., grades) and variables of social background. While the *realistic* and *social* dimensions are positively interrelated with the choice of VET, the *artistic*, *investigative* and *enterprising* dimensions predict the choice of BAC. The *conventional* dimension is the only one positively linked to the choice of FVB. The results are discussed with special attention to segregation between more practical and more theoretical types of interests.

## Introduction

At the end of compulsory school, adolescents in many industrialized countries must make an important and far-reaching decision that strongly influences their future working life (e.g., Hirschi 2011; Koen et al. 2012; Savickas 1999). In several European countries, this decision is mainly between two educational tracks: the vocational and the general (or academic) school tracks (Müller and Shavit 2003). Irrespective of large variations across countries, the general school track often has a better reputation and public perception than the vocational track with regard to occupational outcomes, prestige, income expectations and status preservation (Raffe 2003; Stenström and Lasonen 2000). While recent studies have examined the decision at the end of compulsory school mainly with respect to effects of social background (e.g., Becker and Glauser 2018), in early scientific discussions, the individual interests of the adolescents were described as a key component of track selection (Dewey 1916/1968; Kerschensteiner 1926; Spranger 1923/1965). Early theories especially distinguish between theoretically and practically oriented young people, with each group needing an educational system that provides adequate environments for their personal and skill development (Dewey 1900/2002; Kerschensteiner 1926). Independent of this discussion, the role of interests in educational choices in general has a long tradition in the English-speaking literature (Strong and JR. 1943). In the 1950s in the US, Holland (1959) developed what is probably today’s most dominant interest theory when it comes to occupational choices. His model of career choice is based on the idea of different interest types and corresponding vocational or educational environments (person-environment fit, see Su et al. 2015). The aim of this paper is to link his theory of vocational interests to the choice between general and vocational education at the end of compulsory school and bridge the gap to the idea of a distinction between more theoretically or practically oriented adolescents. This paper examines whether specific vocational interests as defined by Holland (1997) can be seen as predictors for choosing one track or the other since they build different environments (e.g., school vs. company, different types of requirements and examinations, content differences in selectable courses).

For this purpose, in the first step, Holland’s (1997) theory is described and linked to educational choices. Afterwards, the development of segregation between general and vocational education as well as their respective characteristics are explained with a particular focus on the European sector. Since the study was conducted in Switzerland, the Swiss educational system is described, including its specific educational tracks and their relationship to general and vocational education. Next, the tracks are classified in relation to the theory of vocational interests. Based on these remarks, hypotheses are derived and tested via multivariate analyses of variance and multinomial logistic regression analyses. The results and limitations of the study are discussed in the last two chapters.

## Theoretical and Empirical Background

### Linking Interests to Educational Choices: Vocational Interests According to Holland (1997)

The crucial role of interests in vocational choices is described in many career choice theories (e.g., Eccles 1983; Gottfredson 2005; Lent et al. 1994). One of the most popular theories in this context is Holland’s (1959, 1997) model of vocational interests that postulates career choice as a matching process between a person (respective to her or his interests) and an occupation (Low et al. 2005). Holland’s theory is based on several assumptions. The first assumption says that there are six distinct interest types: *realistic*, *investigative*, *artistic*, *social*, *enterprising* and *conventional* (acronym: RIASEC). Interest types are characterized by personal preferences for certain (vocational) activities, skills and attitudes. *Realistic* interests mean a preference for “systematic manipulation of objects, tools, machines and animals”, *investigative* for “systematic, and creative investigation of physical, biological, and cultural phenomena”, *artistic* for “unsystematized activities that entail the manipulation of physical, verbal or human materials to create art forms or products”, *social* for “the manipulation of others to inform, train, develop, cure, or enlighten”, *enterprising* for “the manipulation of others to attain organizational goals or economic gain” and *conventional* for “systematic manipulation of data (e.g., keeping records, filing materials, [...])” (Holland 1997, pp. 21–27). It is assumed that every person has interests in all six dimensions to differing degrees, which build a specific interest profile when combined. The second assumption is that individuals’ environments can be described analogously to the six interest types. Environments are characterized by the activities and tasks that are dominant and typical for a specific occupation, company, field of work, or school type as well as by the people who work and act in them. Vocational interests, as “trait-like preferences to engage in activities, contexts in which activities occur, or outcomes associated with preferred activities” (Rounds and Su 2014, p. 98), have a motivational function and energize goal-striving efforts (Su and Nye 2017). In this sense, the third and most important assumption – the so-called congruence hypothesis (Nauta 2013) – proclaims that people search for environments that fit their own interests and that they avoid or leave environments that do not or just barely fit their interests (Holland 1997). These three assumptions implicitly describe the different demands of the career choice process quite well. On the one hand, adolescents have to explore their personalities, which means developing clarity about their own interests, skills and personality traits. On the other hand, they have to find corresponding occupational or educational fields. Last but not least, the fourth assumption indicates that the interaction between a person’s vocational personality respective to her or his interests and the environment determines the person’s well being and behavior. Thus, a closer fit between the person and environment goes along with better outcomes in job performance, educational achievement, social behavior and satisfaction (Holland 1997). A good person-environment fit is therefore desirable.

Numerous empirical studies have confirmed that Holland’s model applies to a variety of cultures and countries (Bullock et al. 2010; Day and Rounds 1998; Holland 1997; Nauta 2010; Spokane and Cruza-Guet 2005). In addition to the well-documented finding of gender disparities (Ion et al. 2017) with strong differences regarding the so-called “People-Things” dimension (Lippa 2010; Prediger 1982), best represented by the *social* (people: female-dominated) and *realistic* (things: male-dominated) interests (Armstrong et al. 2011; Lippa 2001; Lubinski 2000; Nagy et al. 2010; Proyer and Häusler 2007; Su et al. 2009), the congruence hypothesis has been well examined. The latter focuses not only on vocational (Nauta 2010; Sheu et al. 2010; Sverko and Babarovic 2008; Tracey and Hopkins 2001; Volodina and Nagy 2016) but also on educational choices (e.g., Golle et al. 2019; Marks 2013; Päßler and Hell 2012; Patrick et al. 2011; Usslepp et al. 2020). The main results show that vocational interests are dominant and unique predictors of these choices over and above other (psychological) core variables such as gender, self-efficacy, (cognitive) abilities, school grades or needs and values (Larson et al. 2010; Lent et al. 2003; Lent et al. 2010; Leung et al. 2014; Päßler and Hell 2012; Volodina and Nagy 2016). The meaning of vocational interests has been examined for the transition into tertiary education with regard to college major choices (Care 1996; Larson et al. 2010; Päßler and Hell 2012), for course selection in upper secondary education (Elsworth et al. 1999; Leung et al. 2014; Marks 2013) and for school affiliation at the lower secondary level (von Maurice and Bäumer 2015). Additionally, there are hints that vocational interests discriminate between general educational and vocational or applied learning tracks because both tracks represent different learning environments, go along with different levels of educational achievement standards and provide specific content profiles. However, overall, the state of research regarding this question must be described as rudimentary. The results show that *realistic* interests are mainly related to technical courses that are foremost embedded in vocational education, while *investigative* interests are related to engineering, mathematics and natural science courses that are all mainly embedded in general educational tracks (Golle et al. 2019; Patrick et al. 2011; Stoll 2013). Overall, *investigative* and *artistic* interests are generally associated with a higher level of educational achievement (Stoll and Trautwein 2017) and therefore should be predictive for the academic track. The other interest dimensions are not related to specific educational tracks. *Social* interests are mainly associated with health, physical education and pedagogy, and *enterprising* and *conventional* interests are both linked to economics and business profiles (Ainley et al. 1990; Ainley et al. 1994; Care 1996; Holland 1997; Marks 2013).

In addition to interests, (sociological) studies that try to explain educational choices mostly refer to expectancy-value (Eccles et al. 1993) and rational choice theories (Boudon 1974) in which the decisions of the youth are foremost linked to cost-benefit considerations such as future employment opportunities, status expectations, probability of success, and educational aspirations (for an overview see Becker 2019 or Trebbels 2015). The decision to pursue vocational or general education can also be explained by using these theories (Becker and Hecken 2009). Since, in most countries vocational educational tracks are associated with a worse image or lower prestige than general tracks (Billett 2014), the benefits of these pathways are systematically rated lower than those of general education. However, this does not apply in the same way to the Swiss case, in which Vocational Education and Training is highly regarded (Cattaneo and Wolter 2016; Wolter and Zumbuehl 2017). Since the focus of this paper lies in interests, we will not refer to these theories in detail but will consider significant variables referring to social or migration backgrounds as control variables.

Finally, the choice of track depends on the organization of the educational system, particularly the ways in which qualifications can be obtained for specific occupations. Because this study was conducted in Switzerland, the following two chapters give a brief introduction to the segregation between general and vocational education in general and the Swiss educational system in particular as well as the existing vocational and general educational options. From these explanations, hypotheses are derived regarding the relationship between the interest profiles and the different educational tracks.

### Segregation between General and Vocational Education

The (strong) segregation between general and vocational education in educational systems in Europe and in the Anglo-Saxon countries (e.g., USA, Canada, Australia) has grown historically (Dewey 1916/1968; European Centre for the Development of Vocational Training 2004; Gonon 2009a; Lipsmeier 1966; McClure et al. 1985). For a long time, general education was seen as the only true education (Dewey 1916/1968). It was explicitly separated from technical and practical content, which was considered to have no place in school or education but should be learned “on the job” after one’s school career (Humboldt 1809/1964; Kett 2017). However, at the beginning of industrialization, policy makers, among others, had to establish “a new concept of education [where] [...] for example the natural sciences and ‘realistic’ contents quite generally needed to be re-valued vis-à-vis classical philology” (Gonon 2009b, p. 14). Within this discussion, the supposed contradiction between general and vocational education was called into question, and the two areas were conceived as complementary (Aarkrog 2005; Dewey 1916/1968; Kerschensteiner 1954). With the idea of seeing vocational education as civic education, the former was freed from its image of economic and technical purposefulness and imbued with the notion of broader societal utility (Lipsmeier 1999). As a result, in German-speaking Europe, vocational education gained an increasingly positive reputation – especially in Switzerland (Cattaneo and Wolter 2016; Deissinger and Gonon 2015; Forrer 1998).

Another aspect of the discussion concerns the idea of different types of adolescents who have a higher affinity either for vocational and more practical education or general and more theoretical education (European Centre for the Development of Vocational Training 2013). Kerschensteiner (1926, 279 ff.) addresses this idea in his “law of branching of interests”, where he talks about a diversion of theoretical and practical interests. In his opinion, adolescents at the end of compulsory school are often more interested in practical education, although theoretical interests may branch off from practical interests later in their education or working life. Therefore, he argues that compulsory school should not only pave the way to Baccalaureate Schools but also into the world of work or into vocational schools for those who are not inclined to and do not aspire to the academic path (Gonon 2014; Jackson 2013; Kerschensteiner 1954; english-speaking references: Gonon 2009b; Simons 1966). Equivalent thoughts are formulated by Dewey (1900/2002, 1916/1968) who distinguished between “intellectual interests” and “practical impulses” (1900/2002, p. 26). Later, Spranger (1923/1965, p. 12) also argued that “the scope of the general educational impulse is not as wide for all young people as the higher school would like to assume”. It is more about becoming aware of one’s own preferences, interests and abilities and discovering what one is “destined” to do. Based on that, one chooses the path – academic or vocational – that leads to an appropriate occupation (Hirschi 2011; Parsons 1909). Keeping in mind the arguments of Kerschensteiner (1926), Spranger (1923/1965) and Dewey (1900/2002) along with the focus on different environments in the general and the vocational tracks, Holland’s theory (1997) seems well suited for predicting decisions at the end of compulsory school.

An actual free choice would require that both tracks be seen as equally valuable. There are hints and arguments for “parity of esteem” (Gonon 2008, p. 63) of the two tracks (for Switzerland: Cattaneo and Wolter 2016; Forrer 1998; Strahm et al. 2014) and that they complement each other (Aarkrog 2005; Bowman 1988). However, in many countries in the context of worldwide educational expansion and in the economic literature, general education is seen as more valuable than vocational education, especially with regard to long-term economic effects (Hanushek et al. 2017; Korber and Oesch 2019; Korpi et al. 2003). In fact, in some countries, vocational education is only of marginal importance (Baethge and Wolter 2015; Billett 2014; Gonon 2009a; Strahm et al. 2014).

### The Swiss Educational System

Despite Switzerland’s federal educational structure and its cantonal^1^ differences, a general basic framework can be described (SCCRE 2018). After compulsory school, students can choose between various tracks at the upper secondary level, each of which is clearly assigned to either the general or the vocational educational track. Most representative of the general educational track is the Baccalaureate School (BAC). Overall education in this track is exclusively school-based. The main educational goals of BAC are to prepare young people for university studies (general study ability) and to convey deepened societal responsibility to prepare them for challenging tasks in the society (Eberle and Brüggenbrock 2013). Vocational or specialist training is explicitly excluded from the educational goals of BAC (Federal Council 1995). Instead, the acquisition of vocation-specific qualifications is one of the main purposes of Vocational Education and Training (VET). Education in this context is mainly company-based education supplemented by a small part of school-based education (approximately one day per week). In contrast to the in-depth general education in BAC, vocational education should only provide basic general education that empowers young people to find access to the world of work and to integrate themselves into society (Federal Assembly 2002). In addition to these two tracks, there is a third alternative, namely Federal Vocational Baccalaureate (FVB). It is a hybrid qualification (Deissinger et al. 2013; Graf 2013, 2015) that combines VET (advanced requirements) with deeper general education (Gonon 2013). It therefore links the vocational and general tracks. It is intended to prepare young people for studies at universities of applied sciences and, similar to BAC, for challenging tasks within the economy and society. This education is also mainly company-based education supplemented by much higher proportions of school-based education than VET (approximately three to four times more lessons in school). In contrast to BAC, this education also is vocation specific and focused on the world of work (Federal Council 2009). In 2018, 365,324 students were at the upper secondary level. Apart from regional differences, approximately 20% of the students attended BAC, 54% attended VET and another 10% attended FVB. The numbers show the important position of vocational education (approximately 64%) in Switzerland (SCCRE 2018).

In the first step, the three above-mentioned main educational tracks of the upper secondary level are described in more detail, and the various profiles within the tracks are classified according to Holland’s (1997) RIASEC types based on Bergmann and Eder (2005).

BAC usually lasts four years, and students graduate with the so-called “Matura”. Compared to other European countries, the Swiss Matura plays a special role, because the final certificate of BAC allows unrestricted access to every course of study at any Swiss university (except for medicine, which additionally requires an assessment test) (Eberle and Brüggenbrock 2013). For this reason, there is a particular interest in ensuring the general ability to study and even more the high quality of general education at the end of school, regardless of the canton in which the Matura was obtained (Bonati 2017; Brüggenbrock et al. 2016). These quality demands lead to a relatively low general maturity rate in Switzerland (overall about 22%; Federal Statistical Office 2019b). However, it must be mentioned that the maturity rates vary widely between the cantons (ranging from 13% in Glarus up to 34% in Genf), especially between French-speaking (rather high rates) and German-speaking (rather low rates) cantons. One possible explanation for these variations could be regional differences in the appreciation of vocational education compared with academic education or cantonal differences with regard to selection processes (Eberle and Brüggenbrock 2013). In Switzerland, the differences are discussed with particular regard to questions of educational inequality (discrimination by canton) and with regard to comparable curriculum standards between the cantons (for detailed information see Bonati 2017, Brüggenbrock et al. 2016 or Eberle and Brüggenbrock 2013).

BAC students can choose from different major subjects, which can be categorized as follows (Federal Statistical Office 2018c): Math/Science (chosen by approximately 30% of one BAC cohort; *investigative* environment), Old or Modern Languages (approximately 25%; *artistic* environment), Economics and Law (approximately 24%; *conventional*/*enterprising* environment), Music/Arts (approximately 13%; *artistic* environment) or Pedagogy/Psychology/Philosophy (approximately 7%; heterogeneous, but closest to *social*). Looking at the above dominant profiles and the findings for the relation of the RIASEC dimensions to general education (previous chapter), it can be assumed that *investigative* and *artistic* interests should predict the choice of BAC. The *social* and *realistic* dimensions do not or only rarely find equivalent environments within this profile and should be negatively related to this opportunity. Although there exist adequate environments for the *conventional* and *enterprising* interests, their prediction is less clear, because there are also dominant environments for these interest dimensions in the other tracks, as described below.

In the vocational track, students can choose between the so-called “dual system”^2^ (combination of practical education in a company with theoretical education in a vocational school) and a full-time school model. The dual model is chosen by approximately 80% of Swiss adolescents within the vocational track. In the following, VET refers to the dual system. Overall, more than 230 apprenticeships that can be categorized into 32 occupational fields (Federal Statistical Office 2019c) are available. Depending on the occupation and the requirement level, an apprenticeship lasts from two to four years. The share of apprentices within the different occupational fields is relatively uneven. The top 12 occupational fields of the apprenticeships are concentrated in the commercial (over 30%; *conventional*/*enterprising* environment), the technical and engineering (over 30%; *realistic* environment), and the social and service sectors (approximately 12%; *social* environment). Based on the dominant environments and (again) the findings for the relation of the RIASEC dimensions to vocational education (previous chapter), we can assume that *realistic* and *social* interests should predict the choice of VET. For the *conventional* and *enterprising* dimensions, the prediction is less clear because there are also strong profiles within BAC and the FVB. The *investigative* and *artistic* interests do not find an adequate environment and therefore should be negatively related to this opportunity.

In the early 1990s, the FVB was introduced as an additional opportunity at the upper secondary level. The curriculum in vocational schools for classical VET encompasses 360 to 480 lessons of general education (a half day per week in school), while the respective curriculum of the FVB includes a minimum of 1,440 lessons (State Secretariat for Education, Research and Innovation 2017). The FVB certificate allows adolescents to move on to universities of applied sciences in one of the occupation-related fields of study. The FVB can be attained in two ways: either during (integrated model, two days per week in school, option 1) or after the apprenticeship (consecutive model, one year full-time, option 2). While option 1 was the originally intended and initially the dominant path (two-thirds of FVB students chose this option), option 2 has become increasingly important in recent years (Federal Statistical Office 2019a; Gonon 2013). Therefore, the decision for or against the FVB is often shifted from the end of compulsory school to the end of an apprenticeship. Depending on the occupational field, students are assigned to one of six FVB profiles^3^: technical, commercial, health and social affairs, artistic, scientific, and industrial profiles. The highest shares of FVB students can be found in the commercial (approximately 50%; *conventional*/*enterprising* environment) and technical profiles (approximately 25%; *realistic* environment), while the industrial and scientific profiles have the smallest shares (only 1–2%; *investigative* environment). The health and social affairs profile (*social* environment) was introduced in 2003 (as was the scientific profile) and includes approximately 15% of students today. It is the fastest growing FVB profile of the last 15 years (more than a 400% increase) (Kost et al. 2017). Last but not least, approximately 6% of students choose the FVB within an occupation related to the artistic profile (*artistic* environment). Based on the profile descriptions, it can be said that the *conventional* and *enterprising* environments are well represented within the FVB. Because these interest dimensions also find adequate environments within the other opportunities, no assumptions are formulated for these. Although the technical profile is relatively strong, in absolute numbers compared to VET, it is still small. For this reason, no assumption is formulated for this interest dimension either. The same holds for *social* interests. The *investigative* and *artistic* environments are not well represented and should be negatively related to the choice of the FVB.

### Other Factors Influencing Educational Choice

In addition to interests, a number of other variables play a role in the choice of the educational track in upper secondary education. Looking at the transition from lower to upper secondary level, several disparities are observable. In lower secondary school (age 11/12 to 16/17 years), students are allocated to three different school levels (low, extended, high), depending on their performance in primary school. This stratification already has a significant preallocation effect on the transition to upper secondary education later on (e.g. Becker et al. 2020; Buchmann et al. 2016; Glauser and Becker 2016). While students at the lowest level de facto do not have a chance to attend BAC, those attending the highest level are in general explicitly prepared for this school type, and most of them indeed choose this postcompulsory education after lower secondary school (Becker and Glauser 2018; Glauser and Becker 2016; SCCRE 2018). However, there is still a significant share of students at the extended and high level who decide against BAC, instead choosing VET with or without the FVB (Jäpel 2017; SCCRE 2018). This decision is also dependent on their grades in the main subjects. Although there are cantonal differences, in general, an average grade higher than 4.0 to 4.5^4^ is required to attend the FVB or BAC. Furthermore, independent of school performance, a huge gender gap has existed in the upper secondary level for a long time: as in many other countries, on the one hand, this applies to the choice of the general against the vocational educational track in which female adolescents are overrepresented in the former and male adolescents in the latter (Becker and Glauser 2018; Heiniger and Imdorf 2018; Imdorf et al. 2015). On the other hand, it applies to choices of different occupations within VET in which boys are overrepresented in the technical and girls in the social occupational field (Forster et al. 2016; Hampf and Woessmann 2017; Hanushek et al. 2017). Moreover, disparities of social background as well as native-migrant disparities exist at the transition to and within upper secondary education (Becker and Glauser 2018; Flisi et al. 2016; SCCRE 2018; Wolter and Zumbuehl 2017). Disparities in social background are mainly explained by rational choice and expectancy-value theories as mentioned above. It can be shown that students with lower social status have higher probabilities of choosing vocational educational tracks (Glauser and Becker 2016). This can be explained by differences in school performance and cost-benefit considerations. Students with a lower social background tend to overestimate the costs and underestimate the benefits of general education (and vice versa). Vocational education overall goes along with a lower social status. On the one hand native-migrant disparities can be explained by performance disparities in lower secondary school, on the other hand, they can be explained by differences in aspirations and a gap in information about the educational systems as well as the perspectives or values of the available tracks (e.g., vocational versus general education) (Wolter and Zumbuehl 2017). When basic competencies are controlled, students with a migration background more often aspire to general rather than vocational educational tracks since the latter often have less reputation in the immigrants’ home countries than in Switzerland (Buchmann et al. 2016; Usslepp et al. 2020; Wolter and Zumbuehl 2017).

## Research Question and Hypotheses

Until recently, questions about the choice of either general or vocational education were predominantly answered by focusing on social disparities (Becker and Glauser 2018) and/or economic values over the life course, such as cost-benefit considerations, unemployment risk, image of vocational tracks, and social returns (Becker and Hecken 2009; Hanushek et al. 2017). This study fills a gap in the research with regard to classical career choice theories by focusing on the individual level. From this perspective, it is reasonable to use Holland’s (1997) theory of vocational choices to explain the choice of specific educational or vocational tracks at this stage of the educational career. As shown above, students can choose between different educational tracks that represent environments that differ not only in achievement requirements but also regarding the share of time that has to be spent in school, practical- vs. theoretical-based education and content-related profiles that correspond to specific interest types. Depending on their specific interest profiles, students must answer the question of whether school-based general education with its specific profiles is an adequate environment or whether the alternative way via VET, including the possibility of choosing the FVB as a connecting pathway to higher education, is preferable. This question is faced particularly by high-performing students with the formal opportunity to move on to BAC. Alternatively, it can be asked whether for those high-performing adolescents who do not move on to BAC, vocational interests are the main driver of their decision.

In summary, based on the above assumptions and elaborations, the following hypotheses are derived with regard to the decision for either VET, the FVB or BAC on upper secondary level:

- H1a) The *realistic* dimension is positively related to the decision for VET.
- H1b) The *realistic* dimension is negatively related to the decision for BAC.
- H2a) The *investigative* dimension is positively related to the decision for BAC
- H2b) The *investigative* dimension is negatively related to the decision for VET.
- H3a) The *artistic* dimension is positively related to the decision for BAC.
- H3b) The *artistic* dimension is negatively related to the decision for VET.
- H4a) The *social* dimension is positively related to the decision for VET.
- H4b) The *social* dimension is negatively related to the decision for BAC.
- H5) All six RIASEC dimensions are unique predictors of the decision for VET, the FVB and BAC, even when controlling relevant school variables (attended school level in lower secondary school, recommendation for BAC, grades in German and Mathematics, and perceived person-environment fit with school), individual characteristics (gender, socioeconomic background, and migration background) and canton affiliation.

Because the relationship between the two interest dimensions *enterprising* and *conventional* is less clear, given the adequate profiles of all three tracks and no clear discrimination between the general and vocational track, no hypotheses are formulated for these dimensions. Furthermore, no hypotheses are formulated for the FVB because there are no dominant environments and it is not clearly associated with the general or vocational educational track.

## Method

### Design

To answer the research question and test the hypotheses, we used cross-sectional data from the project “Effects of Selection” (WiSel II – “Wirkungen der Selektion”). WiSell II is a follow-up study of WiSell I, which examined the transition process from primary to lower secondary education. In 2016, students in four German-speaking cantons in Switzerland (Aargau, Basel-Landschaft, Berne, and Lucerne) were asked about their upper secondary educational plans at the end of 9th grade (last year of compulsory school). The participants were mainly surveyed within their classes using a full standardized (online) questionnaire. Students who already participated in WiSel I were surveyed at home. In this case, no data about the class or school affiliation were available.

### Instruments

#### Educational Track

Students were asked about their educational plans after compulsory school. The information provided by the adolescents was evaluated using a teacher questionnaire at the end of grade 9, shortly before the transition into postcompulsory education. In the case of deviations, the information provided by the teachers was used as this information could be considered more valid. The adolescents selected for the analyses were those who intended to do VET (= 1) or to attend the FVB (=2) or BAC (=3).

#### Vocational Interests

Vocational interests were assessed using a short version of the Revised General Interest Structure Test (AIST-R, Allgemeiner Interessen-Struktur-Test - Revision; Bergmann and Eder 2011). The AIST-R is the most frequently used instrument for the measurement of vocational interests in German-speaking Europe (Germany, Switzerland, Austria; Stoll et al. 2017). The six dimensions (*realistic, investigative, artistic, social, enterprising, conventional*) based on Holland’s theory of vocational interests (Holland 1959, 1997) were assessed using five items per dimension. An example of an item of each dimension can be found in the appendix (Table 6). Students were asked to rate their interest in specific tasks on a 5-point Likert scale (*1: not interested at all – 5: very interested)*. All dimensions reached acceptable to good reliability ratings (.73 < *α* < .82).

#### School Variables

*Grades in German and Mathematics* were assessed with self-reports of the students based on the 8th grade final diploma. For this purpose, teachers were asked to bring the final diplomas to their classes on the date of the survey. If they forgot the final diploma or students completed the questionnaire at home, they had to remember their grades. Grades were reported on a scale from 1 to 6, where 1 was the lowest and 6 was the best. Moreover, students reported whether they had a *recommendation for BAC* or not, which was coded dichotomously (0: no, 1: yes). Finally, the *person-school fit* was assessed using an instrument developed by Neuenschwander and Frank (2009) that consists of five items (*Example [translated]: “In my current situation at school…I can use my strengths very well”*) on a 6-point Likert scale (*1: not true at all, 6: absolutely true*). The scale had a good reliability rating (α = .83).

#### Social and Ethnic Background

As an indicator of the social background of the students, the *highest international socioeconomic index of occupational status (HISEI)* (Ganzeboom 2010) was used. For this purpose, the occupations of both parents were first coded with the four-digit ISCO-08-code (International Standard Classification of Occupations, International Labour Organization 2008) and then transformed into the four-digit ISEI-code (Ganzeboom 2010). Finally, the HISEI represented the higher of the two ISEI codes of the parents. Moreover, the *migration background* was assessed with a dummy variable indicating whether a student was born in Switzerland (coded as 1) or not (coded as 0). *Gender* was used as a background variable and was also operationalized dichotomously representing biological sex (0: female, 1: male).

#### School Level in Lower Secondary School

In each of the four examined cantons, lower secondary school can be divided into three *school levels* (low – extended – high). While students in all subjects in Aargau and Basel-Landschaft attend one specific level, in Berne and Lucerne, it is possible that the school level varies across subjects. In this case, the overall school level for the students was coded depending on the school level that most subjects were attending. Furthermore, a (long-term) BAC track in lower secondary school exists in Lucerne, and some students in the canton of Berne had already transferred to BAC after the 8th grade. While all of the prebaccalaureate students in Lucerne moved on to BAC in upper secondary school, in Berne, a substantial share of the BAC students (approximately 25%) moved on to the vocational track (via VET with or without the FVB) after the 9th grade. Hence, this school track was coded as high for Berne, whereas prebaccalaureate students (*n* = 47) were dropped from the analyses for Lucerne (decision already made). This was also the case for students in Aargau who attended the middle school track (*n* = 105) because they did not have a formal opportunity to move on to BAC (Kanton Aargau 2016). Additionally, in all cantons, students in the lowest school track were dropped from the analyses because they did not have a formal opportunity to choose, given formal restrictions.

### Sample

Because the main question of this paper investigated the determinants of the decision among VET, the FVB and BAC, only students who chose one of these three types of education were considered in the analyses. A further condition for the selection of cases was to only examine those students who had attended the medium or highest school level in lower secondary school and obtained an average school grade higher than 4.5 in the subjects German and Mathematics as these were the only students who had the formal opportunity to move on to BAC. The analyzed sample consisted of *N* = 609 students distributed across the four cantons, as shown in Table 1. The share of students per canton approximately represented the distribution of the population of students at the middle and high achievement levels in lower secondary education^5^ (Federal Statistical Office 2018a). Overall, slightly more female (53%) than male students participated in the study, which also reflected the distribution in the population (Federal Statistical Office 2018a). Students were distributed among 63 schools (number of students per school ranged from 1 to 31) and 111 classes (number of students per class ranged from 1 to 17). For 77 students (approximately 13%), there was no information about school and class affiliation available. The mean age was approximately 15.37 years (*Min* = 14, *Max* = 18, *SD* = 0.64).

**Table 1:** Sample description

	*N*		*gender*		*age*			
	total	in %	f (%)	m (%)	*M*	*SD*	*Min*	*Max*
	609	100.0	53.2	46.8	15.37	0.64	14	18
Aargau	135	22.2	56.3	43.7	15.44	0.66	14	18
Basel-Landschaft	76	12.5	50.0	50.0	15.54	0.66	15	17
Berne	219	36.0	56.2	43.8	15.43	0.54	14	17
Lucerne	179	29.4	53.2	46.8	15.17	0.67	14	18

*Note: f = female; m = male; M = mean value; SD = standard deviation; Min = minimum; Max = maximum*

### Analytic Methods

Although missing values in the raw data only had a minor impact on the results (they ranged from 0.7 to 7.3% per variable at the item level), due to cumulative effects in multivariate analyses, missing values were imputed at the item level using the *Fully Conditional Specification Method (FCSM)* in SPSS (IBM Corp 2017; van Buuren 2007). To consider uncertainties associated with imputations, m = 20 imputed data sets were built, and analyses were based on pooled test statistics. Furthermore, the canton affiliation of the students was included in the analysis model by three dichotomous control variables, with Lucerne as the reference canton to take into account nested data structures. It was not possible to control for school or class affiliation because of excessive missing information about these variables (approximately 22%).

First, we tested for differences in vocational interests with regard to specific group affiliation (gender and chosen track in upper secondary school) via multivariate analyses of variance (MANOVA). Because the assumption of equal variance-covariance matrices was not given for any of the six dependent variables (vocational interests), Pillai’s Trace estimator was used because it is most robust against a violation of this assumption (Field 2009). No equality of variances was given for the *realistic* and *conventional* dimensions for the three school tracks. Therefore, a conservative approach was used regarding the significance level so that differences were only reported for a significance level of *p* < 0.01 (Hair et al. 2019). In a second step, the unique predictive validity of vocational interests was tested using stepwise multinomial logistic regression analyses (Long and Freese 2014). Average marginal effects (AME, Long and Freese 2014) estimated in STATA (StataCorp 2017) were used as estimators to compare the different models. In the first model, only vocational interests were examined additionally to canton affiliation. Subsequently, important school variables were included as control variables. In a third step, these school variables were excluded, and variables of social and ethnic background were included as control variables instead. The final (full) model consisted of all variables, vocational interests, and school and background variables. With this procedure, the explanatory power of vocational interests over and above different sets of background and school variables was examined.

## Results

### Descriptive Statistics of the Educational Tracks and Correlations

In the first step, descriptive statistics were used to characterize the adolescents in the different educational tracks (see Table 2). Nearly half of the respondents (44.8%) chose VET, approximately two-fifths (40.2%) chose BAC, and approximately 14% chose the FVB. The overall gender proportion was almost balanced, but it varied significantly between the general educational and vocational tracks (χ^2^(2) = 27.8; *p* < .000, Cramer’s V = .21). While the FVB was predominantly chosen by male adolescents (just above three-fifths) and BAC was predominantly chosen by female adolescents (approximately two-thirds), the gender ratio with regard to VET was roughly balanced. The same held with regard to the overall ratio of the school level attended at the lower secondary level. However, the differences between the three educational tracks were even larger (χ^2^(2) = 222.4; *p* < .000; Cramer’s V = .60) than those for gender. While the proportion of adolescents from the highest school level for VET was approximately 25%, it was 90% for BAC. There was a more balanced relationship between the two school levels for the FVB, where the proportion of adolescents from the highest school level was approximately 60%. Finally, the mean values of the HISEI gave an indication of a medium effect of social background at the transition to the upper secondary level and showed a familiar picture of adolescents in the various tracks differing in social background (F(2,606) = 35.1; *p* < .000; d = .69). While adolescents in BAC had the highest HISEI with an average value of 67.35, adolescents in VET, at 53.40, fell approximately four-fifths of a standard deviation below this value (t(515.6) = 8.10, p < .000). With an average HISEI of 63.62, the adolescents in the FVB were in between, differing significantly from those in VET (t(362) = 4.12, p < .000) but not from those in BAC (t(334) = 1.66, *p* = .244). *P*-values of the post hoc analyses were corrected by the Games-Howell procedure.

**Table 2:** Descriptive statistics for the educational tracks in upper secondary school by gender, school level in lower secondary school and HISEI

			gender		school level		
			female	male	middle	high	HISEI
	n	in %	in %	in %	in %	in %	M (SD)
VET	273	44.8	47.3	52.7	74.4	25.6	53.40 (20.6)
FVB	91	14.9	37.4	62.6	40.7	59.3	63.62 (18.7)
BAC	245	40.2	65.5	35.5	9.4	90.6	67.35 (18.0)
total	609	100.0	53.2	46.8	51.5	48.5	59.28 (20.4)

*Note: VET = Vocational Education and Training; FVB = Federal Vocational Baccalaureate; BAC = Baccalaureate School; HISEI = Highest International Socio-Economic Index of Occupational Status*

Overall, it can be concluded that young people in the three training programs differed significantly in terms of gender (small effect), level of school attendance in lower secondary education (large effect) and social background (moderate effect).

Table 3 shows the correlation matrix of all variables used in the analyses. With regard to the RIASEC dimensions, an interesting pattern occurred for the *realistic* dimension, the dimension theoretically associated with practical content and VET. While all interest dimensions were correlated positively with each other throughout, the *realistic* dimension showed two negative correlations (*artistic* and *social*), and one correlation did not differ significantly from zero (*enterprising*). In addition, the realistic dimension was negatively correlated with the school level attended, recommendation for BAC and the average grade in German and showed no correlation with person-environment fit with the school. All the other interest dimensions showed positive correlations with these variables. The *realistic* dimension was the only dimension that was positively correlated with gender (0: female, 1: male), which is an indicator for the strong gender differences usually found for the RIASEC dimensions. Among the school variables, as expected, there was a high correlation between the recommendation for BAC and the visited school level in lower secondary school. The other variables showed weak correlations, while the perceived fit with the school was only correlated with the average grade in German. The positive correlations with the RIASEC dimensions, on the other hand, were consistently significant. The correlations coefficients of the HISEI variable showed that students with higher interest values and higher values for school variables also showed higher HISEI values (although not all were significantly different from zero). Furthemore, typical gender differences were observable: boys showed lower values in almost all school variables except for grades in Math and, as mentioned above, *realistic* interest, for which positive correlations occurred. The migration status only showed a few weak correlations: Swiss adolescents showed higher values in the *realistic* dimension and lower values in the *enterprising* dimension as well as better grades in German and higher HISEI values.

**Table 3:** Correlation Matrix for the Variables used in the Analyses

	Variable	1	2	3	4	5	6	7	8	9	10	11	12	13	14
	Vocational Interests														
1	Realistic	–													
2	Investigative	**.186**	–												
3	Artistic	**−.128**	**.336**	–											
4	Social	**−.095**	**.322**	**.551**	–										
5	Enterprising	.047	**.233**	**.507**	**.538**	–									
6	Conventional	**.109**	**.219**	**.443**	**.438**	**.734**	–								
	School Variables														
7	School Level (0 = middle)	**−.161**	**.229**	**.146**	**.105**	**.128**	.020	–							
8	Recommendation BAC	**−.161**	**.180**	**.152**	**.127**	**.123**	.033	**.626**	–						
9	Average Grade German	**−.202**	**.109**	**.321**	**.216**	**.139**	**.106**	**.122**	**.233**	–					
10	Average Grade Math	**.066**	**.148**	−.029	**−.108**	**−.066**	−.034	**.136**	**.130**	**−.070**	–				
11	Perceived P-E fit (school)	.013	**.131**	**.152**	**.125**	**.073**	**.086**	.032	.053	**.104**	.064	–			
	Background Variables														
12	HISEI	−.031	**.094**	**.173**	**.103**	**.136**	.054	**.233**	**.215**	**.171**	.061	−.001	–		
13	Gender (0 = female)	**.470**	.003	**−.413**	**−.385**	**−.080**	**−.066**	−.051	**−.093**	**−.300**	**.126**	**−.111**	.001	–	
14	Migration (0 = foreign)	**.137**	−.001	−.038	.001	**−.083**	**−**.021	−.067	−.041	**.125**	−.055	−.043	**.075**	.013	–

*Note: N = 609; Numbers in bold show significant values (p < 0.05)*

### Individual Differences between Students of the Various Educational Tracks

In the second step, it was examined to what extent the students in the various educational tracks differed in terms of their vocational interests. Table 4 shows the descriptive statistics of the students’ vocational interests for the whole sample and separated by gender and educational track.

**Table 4:** Vocational interests of students in the different educational tracks

	***total (n = 609)***	*female (n = 324)*	*male (n = 285)*	*VET (n = 273)*	*FVB (n = 91)*	*BAC (n = 245)*
	M (SD)	M (SD)	M (SD)	M (SD)	M (SD)	M (SD)
REALISTIC	2.44 (0.94)	2.02 (0.70)	**2.92 (0.97)**	**2.72 (1.06)**	**2.60 (0.84)**	*2.08 (0.72)*
INVESTIGATIVE	2.73 (0.99)	2.79 (1.01)	2.80 (1.00)	*2.47 (0.95)*	***2.71 (0.96)***	**3.19 (0.93)**
ARTISTIC	2.63 (0.91)	**3.02 (0.82)**	2.25 (0.87)	*2.39 (0.86)*	*2.52 (0.91)*	**3.00 (0.91)**
SOCIAL	2.76 (1.02)	**3.15 (0.94)**	2.37 (0.94)	*2.65 (1.03)*	*2.58 (1.00)*	**3.01 (0.96)**
ENTERPRISING	2.77 (0.94)	2.85 (0.88**)**	2.70 (0.97)	*2.63 (0.95)*	2.81 (0.90)	**2.93 (0.88)**
CONVENTIONAL	2.43 (0.88)	2.48 (0.85)	2.37 (0.88)	*2.39 (0.92)*	**2.68 (0.93)**	*2.38 (0.76)*

*Note: M = mean value, SD = standard deviation, VET = Vocational Education and Training, FVB = Federal Vocational Baccalaureate, BAC = Baccalaureate School; italic values: significantly smaller value than at least one other group; bold values: significantly higher value than at least one other group; p < 0.01*

In addition to strong gender disparities (Pillai’s trace V = 0.416, F(6, 602) = 61.37, η^2^ = 0.416, see Table 4), there were significant differences regarding the mean values of the RIASEC dimensions with respect to the examined educational tracks (V = 0.355, F(12, 1204) = 21.67, η^2^ = 0.177). While VET or FVB students showed higher interests in the *realistic* dimension than BAC students (F(2, 606) = 33.9, *p* < 0.001, η^2^ = 0.101), the latter showed higher values in the *investigative* (F(2, 606) = 38.1, p < 0.001, η^2^ = 0.112), *artistic* (F(2, 606) = 32.4, p < 0.001, η^2^ = 0.097), *social* (F(2, 606) = 10.73, p < 0.001, η^2^ = 0.034), and *enterprising* (F(2, 606) = 7.3, p < 0.001, η^2^ = 0.023) dimensions. Regarding *investigative* interests, FVB students also showed higher values than VET students. FVB students had higher levels of interest regarding the *conventional* dimension (F(2, 606) = 4.7, *p* < 0.01, η^2^ = 0.015) than did VET and BAC students. As it can be seen from the η^2^ values, the effect sizes ranged from small (*social, enterprising, conventional)* to middle (*realistic, investigative, artistic*), while the overall effect was strong. Regarding the dimensions *investigative*, *artistic*, *social* and *enterprising*, BAC students showed the highest values, whereas interests were much lower for the *conventional* and especially the *realistic* dimensions. However, the analyses showed that in this case the students of the vocational tracks showed higher values both in the *realistic* dimension (VET students) and the *conventional* dimension (FVB students). The values suggested a hierarchical order (except *conventional*) with increasing interest values from VET to BAC for four of the six dimensions and decreasing values for the *realistic* dimension.

### The Prediction of Educational Tracks with Vocational Interests

Table 5 reports the results of the multinomial logistic regression on educational tracks. It became apparent that vocational interests were significant predictors, especially for the decision for VET and BAC and partly for the decision for the FVB. The first model explained 33% of the variance of the dependent variable, reaching 52% in the full model. While school variables also played an important role and additionally explained approximately 17% of the variance (model 2), background variables only helped to explain a further 3.6% of the variance (model 3). The *realistic*, *investigative* and *artistic* dimensions significantly predicted the decision for VET against BAC, whereas the *realistic* dimension was positively related to the decision for VET (negatively related to BAC; H1a and H1b supported), and the *investigative* and *artistic* dimensions were positively related to the decision for BAC (negatively related to VET; H2a and H2b as well as H3a and H3b supported). This held for all four models. Additionally, the *social* dimension was a positive indicator for the decision for VET (H4a supported) and was negatively related to the decision for the FVB. When school variables were controlled, this negative relationship vanished, and there was only a positive prediction for VET (models 2 and 4), while a negative statistical indication for the decision for BAC occurred in model 4 (H4b partly supported). The decision for or against the FVB was not related to one of these three dimensions. The *enterprising* dimension was positively linked to the decision for BAC, even when controlling school and background variables. The negative relationship with the decision for VET only held for the first three models and vanished when both school and background variables were controlled for. There was no linkage to the decision for the FVB. Another strong predictor was the *conventional* dimension, which was the only variable that was predictive for the FVB in the full model. A higher interest in *conventional* tasks was related to a higher probability of choosing the FVB. The decision for BAC was therefore negatively related to higher levels of interest in this dimension. The decision for VET was only weakly related to this dimension, and the relationship did not remain significant when school and background variables were included. The results regarding the cantons showed a consistent pattern. While affiliation with the cantons of Aargau, Berne or Basel-Landschaft – as opposed to affiliation with Lucerne – increased the probability of choosing BAC and lowered the probability of choosing VET, canton affiliation was not relevant for the decision for the FVB. In this context, the strongest relationship could be found for students affiliated with the canton of Aargau (as opposed to Lucerne). Overall, the regression analyses supported H5 because vocational interests were unique predictors for all three educational tracks, even when controlling for relevant school and individual background variables as well as canton affiliation.

**Table 5:** Prediction of educational tracks with vocational interests, school variables and other background variables (multinomial logistic regressions)

	**Model 1**			**Model 2**			**Model 3**			**Model 4**		
	*VET* *(n = 273)*	*FVB* *(n = 91)*	*BAC* *(n = 245)*	*VET* *(n = 273)*	*FVB* *(n = 91)*	*BAC* *(n = 245)*	*VET* *(n = 273)*	*FVB* *(n = 91)*	*BAC* *(n = 245)*	*VET* *(n = 273)*	*FVB* *(n = 91)*	*BAC* *(n = 245)*
***Vocational Interests***												
Realistic	**.103** ***	.023	**−.125** ***	**.067** ***	.024 +	**−.091** ***	**.107** ***	.011	**−.118** ***	**.072** ***	.011	**−.083** ***
Investigative	**−.112** ***	−.008	**.120** ***	**−.056** **	−.015	**.071** ***	**−.114** ***	−.010	**.123** ***	**−.061** ***	−.014	**.076** ***
Artistic	**−.080** **	−.020	**.101** ***	**.070** ***	−.010	**.080** ***	**−.072** **	−.011	**.084** ***	**−.065** **	−.001	**.066** ***
Social	**.056** *	**−.043** *	−.013	**.052** **	−.031	−.021	**.055** **	−.034 +	−.020	**.052** *	−.025	−.027 +
Enterprising	**−.061** *	−.034	**.095** ***	−.033	−.037	**.070** **	−.034	−.042	**.077** **	−.016	−.046 +	**.062** **
Conventional	.050 +	**.095** ***	**−.145** ***	.034	**.095** ***	**−.129** ***	.031	**.092** **	**−.123** ***	.021	**.095** ***	**−.116** ***
***School Variables***												
Highest School Level				**−.249** ***	.013	**.237** ***				**−.221** ***	−.001	**.221** ***
Rcommended for BAC				**−.102** **	.045	**.058** *				**−.099** **	.053	.046
Average Grade German				−.036	.002	.035				−.031	−.007	.038
Average Grade Math				**−.123** ***	**.100** **	.022				**−.111** ***	**.094** **	.017
Perceived P-E Fit (School)				−.033	**−.043** *	**.076** ***				−.036 +	**−.042** *	**.078** ***
***Background Variables***												
Male							−.016	.060 +	−.044	−.017	.058 +	−.041
SES							**−.005** ***	.001	**.003** ***	**−.003** ***	.001	**.002** *
Swiss							.058	.025	−.083 *	.036	.038	**−.074** *
***Control for Cantons*** ^***a***^												
Aargau	**−.514** ***	**.092** *	**.422** ***	**−.248** ***	.010	**.238** ***	**−.450** ***	.076 +	**.373** ***	**−.218** ***	−.005	**.223** ***
Bern	**−.141** ***	.017	**.124** **	**−.197** ***	.029	**.168** ***	**−.119** ***	.014	**.105** **	**−.170** ***	.018	**.152** ***
Basel-Landschaft	**−.248** ***	−.031	**.279** ***	**−.262** ***	−.023	**.285** ***	**−.227** ***	−.034	**.261** ***	**−.236** ***	−.035	**.271** ***
*Pseudo-R* ^*2*^ *(Mc Fadden)*	.330			.499			.366			.521		
*N*	609			609			609			609		

Note: Average Marginal Effects; robust standard errors; + p < 0.10, * p < 0.05, ** p < 0.01, *** p < 0.001; a: Reference is Lucerne; VET = Vocational Education and Training, FVB = Federal Vocational Baccalaureate, BAC = Baccalaureate School

## Discussion and Conclusion

The aim of this paper was to examine the importance of vocational interests in the educational decisions of students at the end of compulsory school in Switzerland. A special focus was placed on the decision between general and vocational education because the opportunities at the upper secondary level (BAC, FVB, VET) are representative of either of these two tracks. First, descriptive and bivariate analyses show strong signs of differences in the vocational interests of students in the various tracks, whereby several confounding effects with respect to other background and school variables can be observed. In addition, multivariate analyses of variance and a multinomial logistic regression were used to examine the differences in vocational interests while controlling these other variables.

All hypotheses could be (at least partly) confirmed by the results, showing the crucial role of vocational interests over and above school variables, variables of social background and cantonal affiliation. This especially holds for the interrelation of interests and the decision for VET against BAC. In contrast, there are almost no interrelations between the decision for the FVB and the different interest dimensions. This is an indicator that the FVB lies between the two other educational programs (Jäpel 2017) and therefore between the general and vocational educational track. Only the *conventional* dimension showed positive interrelations with this track and was negatively correlated with the general school track BAC. The reasons for this result may be due to the different profiles of the FVB and the respective share of students choosing the FVB during apprenticeship (option 1). The *conventional* interest dimension can, by definition, strongly refer to the commercial profile (Bergmann and Eder 2005; Holland 1997). The commercial profile in the FVB is by far the largest (approximately 48% of all FVB students, Federal Statistical Office 2018b), and it is the profile with the highest share (approximately 70%) of students doing the FVB during apprenticeship instead of afterwards (Kaiser et al. 2013). It seems plausible that especially with respect to the commercial occupational field, interests already have an impact at the end of the lower secondary level. For the other profiles, interests that are positively associated with VET (*realistic*, *social*) are probably particularly important for the decision for VET itself regardless of whether the FVB is added. As mentioned, the decision for or against the FVB is often shifted to the end of apprenticeship in these profiles. This is also a further argument for the result that vocational interests mainly discriminate between BAC and VET. In summary, the results indicate that at this point in time, interests are not the main driver for the decision for the FVB but discriminate very well between general and vocational education overall. For young persons, the environments of VET and the FVB probably seem equal in terms of interest satisfaction. Therefore other concerns likely come into play regarding the decision for the FVB (e.g., cost-benefit considerations, educational aspirations).

The profiles provided at BAC can help to further interpret the results. While there is de facto no profile that serves the *realistic* interest types and only one small and heterogeneous profile that builds an adequate environment for *social* types (pedagogy-psychology-philosophy), these two types find the most adequate, important, and traditional profiles in the vocational segment (technical apprenticeship and crafts as well as health and social affairs). This is a plausible explanation for the positive interrelation with VET for these types. Having high values in the *enterprising* dimension raises the probability of attending BAC in upper secondary education. Interests in the *enterprising* dimension are related to leading positions in the economy. Such positions are generally associated with higher educational attainment (Golle et al. 2019) and are mainly restricted to university degrees. This seems especially plausible because the standard path to universities is to go through BAC (SCCRE 2018).

The results confirm the hypothesis that the different educational tracks serve different interest types. Bivariate analyses show large variations in vocational interests with respect to the different tracks, especially between BAC (general) and VET (vocational) as well as the FVB. On the one hand, this can be justified in the different profiles provided within the tracks and the suggestion that for some occupational fields, there is no truly equivalent environment provided within the general educational track (e.g., no profile that serves the *realistic* type in BAC). On the other hand, it must be mentioned that the general and vocational tracks represent completely different environments that are characterized overall by differences in the share of time spent in school and the share of occupation-specific practical content versus occupation-wide theoretical content. The embedded profiles within these tracks did not emerge by chance; they have grown over time, and equivalent profile-related differences between the general and the vocational system can be found in many countries worldwide (European Centre for the Development of Vocational Training 2004; McClure et al. 1985; Stenström and Lasonen 2000). These differences are also often reflected within the different types of vocational interests (Holland 1997). The decision for VET and BAC and therefore for the general or vocational educational track is not only a question of school performance, social background or school affinity but is also essentially an interest-driven decision. Thus, the results relativize the hierarchical order of the three educational tracks, at least for high-performing adolescents; for this reason, it is essential that adolescents are able to understand their own interests. Adolescents should use opportunities to clarify their abilities and interests, which can help them in finding an adequate environment for their further educational or occupational career. Therefore, the results highlight the special role of career counseling at this stage of life. With this in mind, the parity in the reputations of the educational tracks appears to be relevant because it is not desirable that, for example, a young person would forego her or his preferred educational track due to lack of financial prospects. Consequently – probably less so in Switzerland than in other countries – from an educational point of view, it has to be asked whether “there is a need for action with regard to possible perspectives for adolescents who have their strengths more in the context of practical competencies instead of mainly the classical subjects of general education. So that the vocational education gets a high quality orientation and therefore can foster and exploit different performance capacities than the general education does. This has to be discussed not only in educational policy but also with regard to economical education” (Alexander and Pilz 2004, p. 757). Although the study was conducted in Switzerland and therefore results refer to a specific educational system, it can be argued that the systematic differences between general and vocational education are transferable to other countries. Holland’s (1997) vocational interest structures and individual differences have been examined in many cultures and countries. It is reasonable to assume that these differences call for diverse environments that adequately meet the interests of young people. Independent of the detailed organization of the educational system, every system shows a segregation between general and vocational education including specific (practical or theoretical) profiles. If the environments are comparable, the same interest types are predictive for general (e.g., investigative) and vocational (e.g., realistic) education, as shown here. However, more empirical studies are needed to substantiate this issue.

## Limitations and Outlook

The study has several constraints. First, although from a theoretical point of view causality can be reasonably argued, there is a strong restriction in the evidence due to the cross-sectional design of the analyses. Whenever causality is indicated, this has to empirically be treated as pure correlation. Furthermore, in a cross-sectional design, the effects tend to be overestimated. This must be considered when interpreting the results. Another restriction stems from the point in time at which the survey took place. At the end of the 9th grade, the decision about further education had already been made by the vast majority of young people for some time (partly more than one year). It can be assumed that this choice influenced the way adolescents answered the interest scales because they probably tended to justify their past decisions. Again, this is an argument for being cautious with propositions of causality and effect sizes. A possible approach for future studies would be to systematically survey the interests of adolescents longitudinally in lower secondary school (or even earlier), and to examine interest development among adolescents with regard to the career choice process. In particular, the role of vocational interests with respect to specific career choice actions (internships, counseling, etc.) would be of special interest. Although we considered numerous variables, due to theoretical reasons we did not focus on information concerning regional or societal variations such as job opportunities, economic situation, labor market conditions or regional school density. Nevertheless, the choice processes are also influenced by regional opportunity structures (Glauser and Becker 2016). This could be an interesting question for future research. Furthermore, although nested data structure was controlled for to make robust statements on the individual level, no variables on class, school or canton level were formulated as predictors in order to observe the effects of structural variables. Again, future research should implement these structural variables to provide further information on these levels. The transferability of the results is limited due to characteristics of the sample, which was restricted to high-performing adolescents who had a formal opportunity to decide for or against BAC and FVB. For this reason, no statements can be made about low-performing young people. Further studies could consider the role of vocational interests for this group of students with regard to different educational tracks that were not the focus of this paper (different levels in VET, bridge years and so on). However, despite this restriction, there might still be a share of adolescents within the sample that had no opportunity to move on to BAC. Due to differing transfer conditions between the cantons that are often caused by varying levels of average grades with different weights for various subjects, it was not possible to calculate a dummy variable that more reliably filtered persons who had the opportunity to access BAC based on these data. This is also a limitation because the sample selection only considered grades in German and Mathematics. Because of data restrictions, other subjects were not considered that are also relevant for the transition recommendation (foreign language, natural sciences, etc.). Overall, more empirical studies are needed to examine all the different aspects that contribute to the educational transition process of young people and create a more holistic picture. However, our results show that it is important to include vocational interests in this future research.

## Appendix

**Table 6:** Example Items of the General Interest Structure Test of Bergmann and Eder (2011) Question: How much are you interested in the following activities?

Interest-Dimension	German	English [translated]
Realistic	Mit Maschinen oder technischen Geräten arbeiten.	Working with machines or technical equipment.
Investigative	In einem Versuchslabor Experimente durchführen.	Carrying out experiments in an experimental laboratory.
Artistic	Geschichten oder Reportagen schreiben.	Writing stories or articles.
Social	Andere Personen betreuen oder pflegen.	Caring for other persons.
Enterprising	Ein Geschäft oder Unternehmen führen.	Running a business or enterprise.
Conventional	Eine Buchhaltung führen.	Doing accounting.

*Note: A 5-point Likert scale was used for rating (1: not interested at all – 5: very interested). Translation by the authors*

## Footnotes

1 The federal states in Switzerland are called “cantons”.

2 The expression “dual system” is actually just an example of this kind of education and especially refers to the German system. A more official translation would be “dual-track variety” (State Secretariat for Education, Research and Innovation 2019, p. 4) or “alternance training” (Batzel 2016, p. 5). However, to our knowledge, the most commonly used expression in international contexts is the “dual system”.

3 In a 2009 revision, the six profiles were changed to five broader directions that mainly correspond to five of the six old profiles: Technology/Architecture/Life Sciences, Business and Services, Health and Social Affairs, Design and Arts, Nature/Landscape and Food. The Industrial profile does not exist anymore. The new profiles were finally introduced in 2016.

4 In Switzerland, school grades range from 1 (lowest) to 6 (highest).

5 There are slight distortions, mainly due to the exclusion of specific subsamples (see previous chapter).
